# Combined antiretroviral therapy with low- or normal-protein, high-calorie diets appears to induce significant deleterious electrocardiographic changes in a rodent model

**DOI:** 10.1590/1414-431X2026e14744

**Published:** 2026-04-17

**Authors:** B.M. Chege, P.W. Mwangi, C.G. Githinji, F. Bukachi

**Affiliations:** 1 School of Health Sciences, Dedan Kimathi University of Technology, Nyeri, Kenya; 2 Department of Human Anatomy and Medical Physiology, Faculty of Health Sciences, University of Nairobi, Nairobi, Kenya

**Keywords:** Cardiac electrocardiographic, Calorie-dense with low protein, Calorie-dense with normal protein, Cardiac fibrosis, Combined antiretroviral therapy, Tesamorelin

## Abstract

The introduction of combination antiretroviral therapy (cART) has significantly reduced AIDS-related morbidity and mortality. However, the prevalence of age-associated comorbidities, particularly cardiovascular diseases (CVD), has increased, becoming a leading cause of mortality in people living with HIV. This study investigated the interaction between cART regimens and dietary composition on electrocardiographic (ECG) parameters and myocardial histopathology. A total of 120 weanling Sprague Dawley rats were allocated to one of three diets for 15 weeks: normal chow, a calorie-dense low protein (CDLP) diet, or a calorie-dense normal protein (CDNP) diet. Each dietary group was then subdivided into four treatment groups for a further 9 weeks: a standard group (normal saline), Test group 1 (dolutegravir (DTG) plus tesamorelin), Test group 2 (DTG only), and a positive control (classical cART regimen). ECG recordings and histological assessments were performed at week 24. Significant intergroup variations in ECG indices were observed, including Q, R, S, and T wave amplitudes, PR interval, QRS duration, ST height, and QTc (all P<0.0001). Myocardial fibrosis (P<0.0001) was evident in animals from the TG2 (DTG only) and PC (classical regimen) groups maintained on CDLP and CDNP diets. These findings demonstrated that CDLP and CDNP diets, combined with DTG-based or classical cART regimens, exerted deleterious cardiac effects, promoting myocardial fibrosis that disrupts normal electrical conduction and may predispose to arrhythmogenesis. Tesamorelin prevented these effects, implicating growth hormone pathway dysfunction in the underlying pathology.

## Introduction

The occurrence of high-calorie malnutrition among individuals who are HIV-positive receiving combination antiretroviral therapy (cART) is widespread in Sub-Saharan Africa (SSA) (1). High-calorie malnutrition correlates with various changes in body composition capable of inducing cardiac abnormalities such as arrhythmias, cardiomyopathy, heart failure, and sudden death (2).

Individuals who have HIV on cART face an increased likelihood of cardiovascular disease (CVD), with the incidence of sudden cardiac mortality in this population potentially reaching up to 4.5 times that of the uninfected population (3). Protease inhibitors and non-nucleoside reverse transcriptase inhibitors, such as efavirenz and rilpivirine, have been implicated in coronary heart disease and QTc elongation, consecutively. Protease inhibitors and non-nucleoside reverse transcriptase inhibitors like efavirenz and rilpivirine, have been linked to ischemic heart disease and QTc prolongation, respectively (4,5). HIV infection is independently associated with the presence of ST/T-wave abnormalities and sinus tachycardia (6).

Tesamorelin, an analog of growth hormone-releasing factor, has demonstrated efficacy in reducing visceral adiposity and ameliorating cardiovascular risk parameters in individuals with HIV receiving cART (7). Although not specifically developed for the treatment of CVD, the capacity of tesamorelin to reduce excess visceral abdominal adiposity, a well-established risk factor for CVD in this population, indicates a potential role in CVD prevention (8).

This study explored the relationship between diet, cART regimens, and their effects on ECG and cardiac histology.

## Material and Methods

### Diet preparation

The diets were specially designed and manufactured by Unga Group Limited (Kenya), a local commercial manufacturer, in the concentrations listed in Table 1.

**Table 1:** Dietary formulation of the different diets.

Calorie content (kcal/100g)	Normal chow	Calorie-dense with normal protein (CDNP)	Calorie-dense with lowprotein (CDLP)
Fat	4.8	36	36
Protein	17.1	17.1	6
Complex carbohydrates	34.6	42	42
Sucrose	5.3	20	20

### Animal selection, grouping, and experimental treatment

A total of 120 male Sprague-Dawley rats, weighing approximately 140-150 g, were obtained from the Department of Pharmacognosy, University of Nairobi. They were housed together in the animal house within the Faculty of Health Sciences, where ambient conditions were maintained at a room temperature of 24±2°C, with humidity between 35 and 55%, and a 12-h light/dark cycle.

Over a fourteen-day acclimatization period preceding the study, the animals familiarized themselves with both the experimenter and their environmental surroundings. The experimental procedures were conducted in two phases: the initial phase involved evaluating the obesogenic properties of three distinct diets while the second phase focused on investigating the impacts of various treatments on these diets.

In the initial phase, the rats were randomly assigned to three diet groups (n=40/group) and received either normal chow, calorie-dense normal protein (CDNP), or calorie-dense low protein (CDLP) diets for 15 weeks. Throughout the study, all animal groups had *ad libitum* access to food and water. In the second phase, each group underwent further randomization into four treatments subcategories at week 16, (n=10): Standard group (SG): normal saline; Test group 1 (TG 1) - (DTG regimen + tesamorelin): tenofovir disoproxil fumarate (TDF) + lamivudine (3TC) + dolutegravir (DTG) + tesamorelin; Test group 2 (TG 2) - (DTG regimen only): tenofovir disoproxil fumarate (TDF) + lamivudine (3TC) + dolutegravir (DTG); Positive control (PC) - (classical regimen): zidovudine (AZT) + lamivudine (3TC) + atazanavir/ritonavir (ATV/r).

The treatments were given each day from 2:00 PM to 3:00 PM by oral gavage for 9 weeks. The drug doses were normalized by body weight/surface area from rats to humans following the formula by Nair and Jacob (9): HED (mg/kg) = Animal dose (m/kg) × (Animal Km / Human Km), where HED is Human Equivalent Dose and Km is the correction factor; rat Km is 6.2 and human Km is 37.

The rats' weights were measured weekly using a standard laboratory scale. (Ohaus^®^, China, SJX6201N/E scout portable scale).

### Electrocardiographic recordings

Electrocardiographic (ECG) recordings were conducted at the end of the experiment one day prior to euthanization. The rodents were administered sedative intraperitoneal (*ip*) injection of ketamine hydrochloride (75 mg/kg) and midazolam (2.5 mg/kg) prior to the ECG recording. Electrodes (SKINTACT^®^ Code T601, Leonhard Lang GmbH, Austria) with gel-coated pads were placed on the paws with each animal having been placed on a heat pad for the entire duration of the ECG recording. The neutral electrode was placed on the right front paw, the positive electrode was placed on the left back paw, and the negative electrode was placed on the right front paw for the lead II ECG recording. ECG was recorded using the Powerlab^®^ data acquisition apparatus (ML865™ AD instruments, Australia). The default settings for rat ECG were: band-filter of 10-50 Hz and a voltage range of 2 mV. ECG data were analyzed automatically using software (GraphPad Prism^®^ version 8.0, USA). Data analysis results were verified manually to ensure interpretation accuracy.

The following ECG features were extracted from the standard lead II recordings: P wave amplitude, Q wave amplitude, R wave amplitude, T wave amplitude, R-R interval, heart rate, QRS duration, ST segment amplitude, and QT interval. The QT interval was corrected for heart rate based on the Hodges formula: QT_C_=QT+1.75 (heart rate-60) (10).

### Myocardial fibrosis

Samples from all experimental groups underwent fixation and paraffin embedding. Subsequently, 4-μm-thick serial sections were stained using Masson's trichrome stain. Cardiomyocytes appeared red, while fibrotic areas were stained blue. Fibrosis levels were assessed at ×400 magnification using an Olympus DP71 microscope (Japan) connected to a video camera. Scores were calculated using ImageJ™ software (NIH, USA) as the fibrotic area score relative to the total area of each microscopic field. Ten microscopic fields per sample were analyzed.

### Ethical approval

The consent to carry out the study was obtained from the Biosafety, Animal Care and Use Committee of the Department of Veterinary Anatomy and Physiology at the University of Nairobi (FVM BAUEC/2022/354), adhering to the guidelines outlined in the National Institutes of Health (NIH) Guide for the Care and Use of Laboratory Animals.

### Statistical analysis

Experimental data are reported as means±SE and were analyzed using one-way ANOVA followed by Tukey's test for multiple comparisons. Kruskal Wallis with Dunns' *post hoc* test was used to analyze fibrosis scores. The data analysis was done using GraphPad Prism^®^ (USA) version 8.0.1, and the results were deemed statistically significant when P≤0.05.

## Results

### Electrocardiographic measurements

#### Normal chow

**Figure 1:**
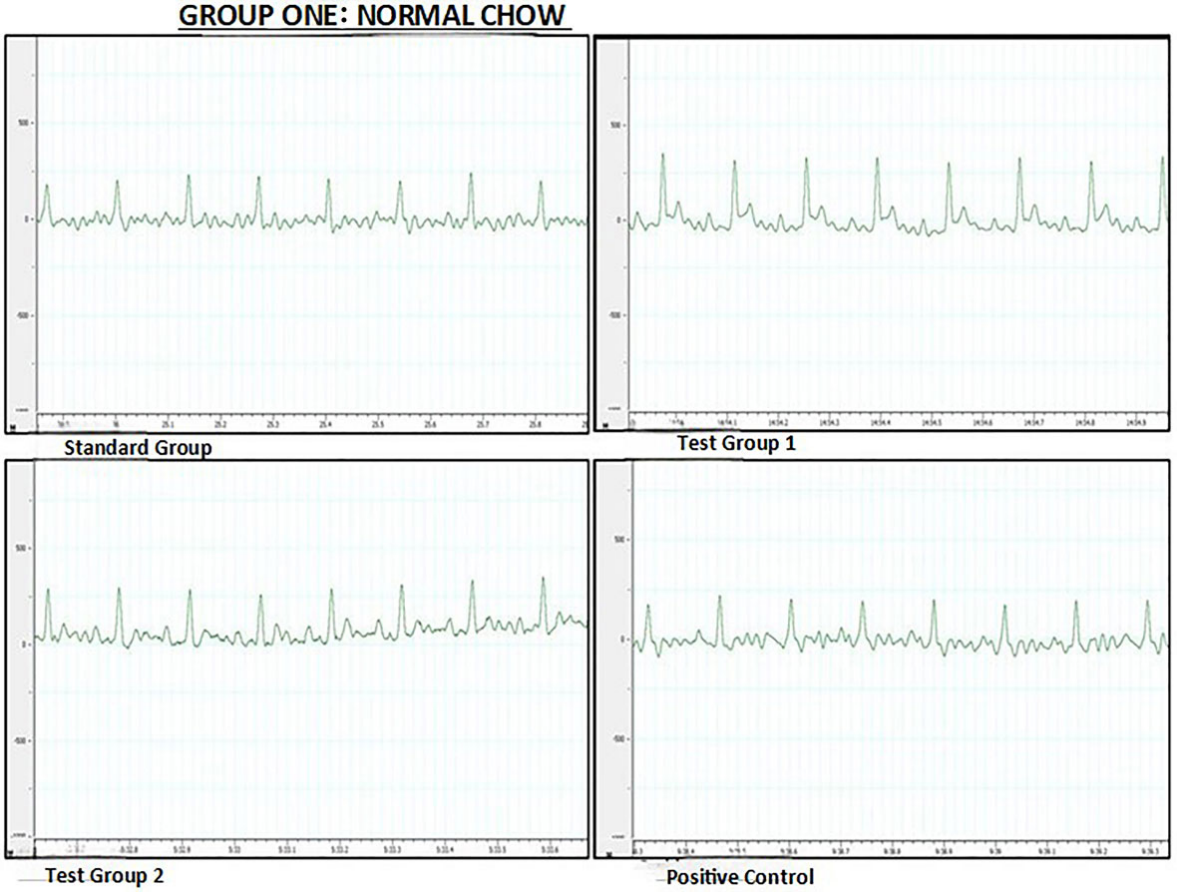
Representative electrocardiographic tracings of the normal chow diet group during the treatment phase. Standard group (SG): normal saline. Test group 1 (TG 1) - (DTG regimen + tesamorelin): Tenofovir disoproxil fumarate (TDF) + lamivudine (3TC) + dolutegravir (DTG) + tesamorelin. Test group 2 (TG 2) - (DTG regimen only): tenofovir disoproxil fumarate (TDF) + lamivudine (3TC) + dolutegravir (DTG). Positive control (PC) - (classical regimen): zidovudine (AZT) + lamivudine (3TC) + atazanavir/ritonavir (ATV/r).

Heart rate (HR) and all ECG parameters were similar among the four experimental groups. ECG measurements and tracings for the normal chow diet group at the end of the treatment phase are shown in Table 2 and Figure 1, respectively.

**Table 2:** Electrocardiographic measurements for the normal chow diet group at the end of the treatment phase.

	Groups				P-value
	SG	TG 1	TG 2	PC	
Heart rate (bpm)	365.40±3.84	368.90±4.06	374.40±4.61	377.10±2.03	0.1353
Q wave amplitude (mV)	-0.01794±0.001489	-0.01820±0.001443	-0.01813±0.001932	-0.01923±0.001575	0.9431
R wave amplitude (mV)	0.1598±0.005626	0.1576±0.005456	0.1639±0.006826	0.1657±0.005109	0.8447
S wave amplitude (mV)	-0.02528±0.001748	-0.02513±0.001250	-0.02546±0.000905	-0.02543±0.0008646	0.9975
T wave amplitude (mV)	0.005380±0.0001718	0.005400±0.0002539	0.00561 ±0.000138	0.005530±0.0001521	0.7888
PR interval (s)	0.04652±0.001875	0.04881±0.001505	0.05036±0.002165	0.04914±0.001258	0.4745
QRS duration (s)	0.02227±0.001134	0.02331±0.001608	0.02572±0.000944	0.02649±0.001284	0.0790
ST height	0.1451±0.001523	0.1459±0.0009414	0.1465±0.001428	0.1492±0.0002058	0.0790
QT interval (s)	0.05624±0.0007748	0.05672±0.001754	0.05709±0.000645	0.05883±0.0002751	0.3172
QTc interval (s)	0.5919±0.006387	0.5973±0.007259	0.6072±0.007713	0.6137±0.003297	0.0901

Data are reported as means±SE (ANOVA). Standard group (SG): normal saline; Test group 1 (TG 1) - (DTG regimen + tesamorelin): Tenofovir disoproxil fumarate (TDF) + lamivudine (3TC) + dolutegravir (DTG) + tesamorelin; Test group 2 (TG 2) - (DTG regimen only): tenofovir disoproxil fumarate (TDF) + lamivudine (3TC) + dolutegravir (DTG); Positive control (PC) - (classical regimen): zidovudine (AZT) + lamivudine (3TC) + atazanavir/ritonavir (ATV/r).

#### Calorie dense with normal protein

Significant variations in heart rate were observed among the four experimental groups. Post hoc statistical analysis, identified significant differences between SG and TG 2, SG and PC, TG 1 and TG 2, and TG 1 and PC (all P<0.0001).

All wave amplitudes and ECG parameters differed significantly among the four experimental groups. *Post hoc* statistical analysis identified significant differences between SG and TG 2, SG and PC, TG 1 and TG 2, and TG 1 and PC in all parameters (all P<0.0001).

**Figure 2:**
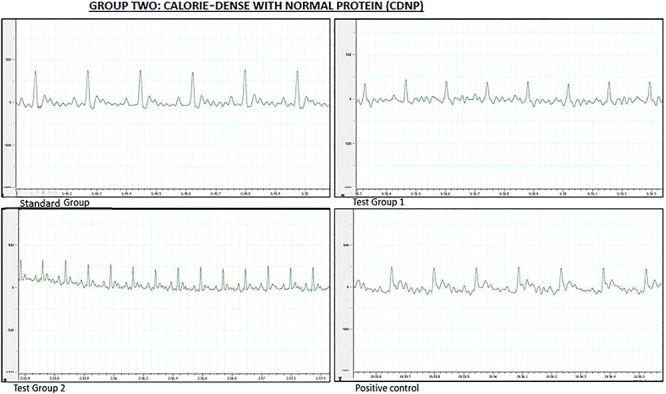
Representative electrocardiographic tracings of the calorie-dense with normal protein diet group during the treatment phase. Standard group (SG): normal saline. Test group 1 (TG 1) - (DTG regimen + tesamorelin): Tenofovir disoproxil fumarate (TDF) + lamivudine (3TC) + dolutegravir (DTG) + tesamorelin. Test group 2 (TG 2) - (DTG regimen only): tenofovir disoproxil fumarate (TDF) + lamivudine (3TC) + dolutegravir (DTG). Positive control (PC) - (classical regimen): zidovudine (AZT) + lamivudine (3TC) + atazanavir/ritonavir (ATV/r).

ECG measurements and tracings for the CDNP diet group at the end of the treatment phase are shown in Table 3 and Figure 2, respectively.

**Table 3:** Electrocardiographic measurements for the calorie-dense with normal protein diet group at the end of the treatment phase.

	Groups				P-value
	SG	TG 1	TG 2	PC	
Heart rate (bpm)	480.40±4.13	481.50±2.28	502.50±2.37	501.20±2.78	<0.0001
Q wave amplitude (mV)	-0.01668±0.0002468	-0.01656±0.0003582	-0.0131±0.0005171	-0.01366±0.0002715	<0.0001
R wave amplitude (mV)	0.3043±0.002996	0.3005±0.005338	0.4039±0.006058	0.4057±0.005925	<0.0001
S wave amplitude (mV)	-0.02099±0.0005063	-0.02112±0.0004643	-0.01348±0.0009116	-0.01383±0.0007683	<0.0001
T wave amplitude (mV)	0.01305 ±0.0008891	0.01451±0.0006208	0.01947±0.0002196	0.01934±0.0001887	<0.0001
PR interval (s)	0.05647±0.001881	0.06145±0.001549	0.08517±0.001259	0.08510±0.001458	<0.0001
QRS duration (s)	0.03035±0.001104	0.03062±0.001222	0.06853±0.003010	0.07201±0.004857	<0.0001
ST height	0.1461±0.0006387	0.1466±0.0009851	0.1530±0.001010	0.1527±0.001835	<0.0001
QT interval (s)	0.06010±0.0007289	0.06099±0.001049	0.07418±0.001899	0.07662±0.001991	<0.0001
QTc interval (s)	0.7958±0.007462	0.7986±0.004072	0.8481±0.005216	0.8486±0.004693	<0.0001

Data are reported as means±SE (ANOVA). Standard group (SG): normal saline; Test group 1 (TG 1) - (DTG regimen + tesamorelin): Tenofovir disoproxil fumarate (TDF) + lamivudine (3TC) + dolutegravir (DTG) + tesamorelin; Test group 2 (TG 2) - (DTG regimen only): tenofovir disoproxil fumarate (TDF) + lamivudine (3TC) + dolutegravir (DTG); Positive control (PC) - (classical regimen): zidovudine (AZT) + lamivudine (3TC) + atazanavir/ritonavir (ATV/r).

#### Calorie-dense with low protein

Significant variations in heart rate were observed among the four experimental groups. *Post hoc* statistical analysis using Tukey's multiple comparisons test identified significant differences between SG and TG 2, SG and PC, TG 1 and TG 2, and TG 1 and PC (all P<0.0001).

**Figure 3:**
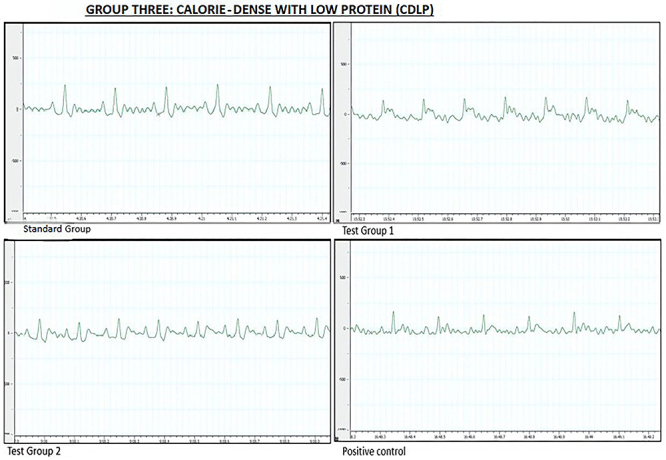
Representative electrocardiographic tracings of the calorie-dense with low protein diet group during the treatment phase. Standard group (SG): normal saline. Test group 1 (TG 1) - (DTG regimen + tesamorelin): Tenofovir disoproxil fumarate (TDF) + lamivudine (3TC) + dolutegravir (DTG) + tesamorelin. Test group 2 (TG 2) - (DTG regimen only): tenofovir disoproxil fumarate (TDF) + lamivudine (3TC) + dolutegravir (DTG). Positive control (PC) - (classical regimen): zidovudine (AZT) + lamivudine (3TC) + atazanavir/ritonavir (ATV/r).

All ECG parameters differed significantly among the four experimental groups. ECG measurements and tracings for the CDLP diet group at the end of the treatment phase are shown in Table 4 and Figure 3, respectively.

**Table 4:** Electrocardiographic measurements for the calorie-dense with low protein diet group at the end of the treatment phase.

	Groups				P-value
	SG	TG 1	TG 2	PC	
Heart rate (bpm)	505.40±4.68	510.50±3.89	554.80±3.87	567.80±3.07	<0.0001
Q wave amplitude	-0.007456±0.001088	-0.007436±0.000875	-0.001580±0.000232	-0.001766±0.0003555	<0.0001
R wave amplitude	0.5019±0.004641	0.4981±0.006261	0.7109±0.007319	0.7169±0.007429	<0.0001
S wave amplitude	-0.009730±0.0008803	-0.01099±0.0002826	-0.1229±0.003098	-0.1293±0.002612	<0.0001
T wave amplitude	0.02049±0.0005986	0.02071±0.0004180	0.02854±0.0009634	0.02934±0.001670	<0.0001
PR interval	0.07930±0.001599	0.08428±0.001995	0.1535±0.008574	0.1616±0.01498	<0.0001
QRS duration	0.05616±0.0008568	0.06085±0.001980	0.08980±0.001757	0.09275±0.001427	<0.0001
ST height	0.1570±0.0007621	0.1573±0.0004957	0.1635±0.001220	0.1641±0.001241	<0.0001
QT interval	0.06994±0.001388	0.07084±0.001001	0.08586±0.001191	0.08563±0.001737	<0.0001
QTc interval	0.8494±0.008061	0.8593±0.006817	0.9322±0.01173	0.9410±0.007578	<0.0001

Data are reported as means±SE (ANOVA). Standard group (SG): normal saline; Test group 1 (TG 1) - (DTG regimen + tesamorelin): Tenofovir disoproxil fumarate (TDF) + lamivudine (3TC) + dolutegravir (DTG) + tesamorelin; Test group 2 (TG 2) - (DTG regimen only): tenofovir disoproxil fumarate (TDF) + lamivudine (3TC) + dolutegravir (DTG); Positive control (PC) - (classical regimen): zidovudine (AZT) + lamivudine (3TC) + atazanavir/ritonavir (ATV/r).

### Histology for left ventricular muscle

#### Normal chow

**Figure 4:**
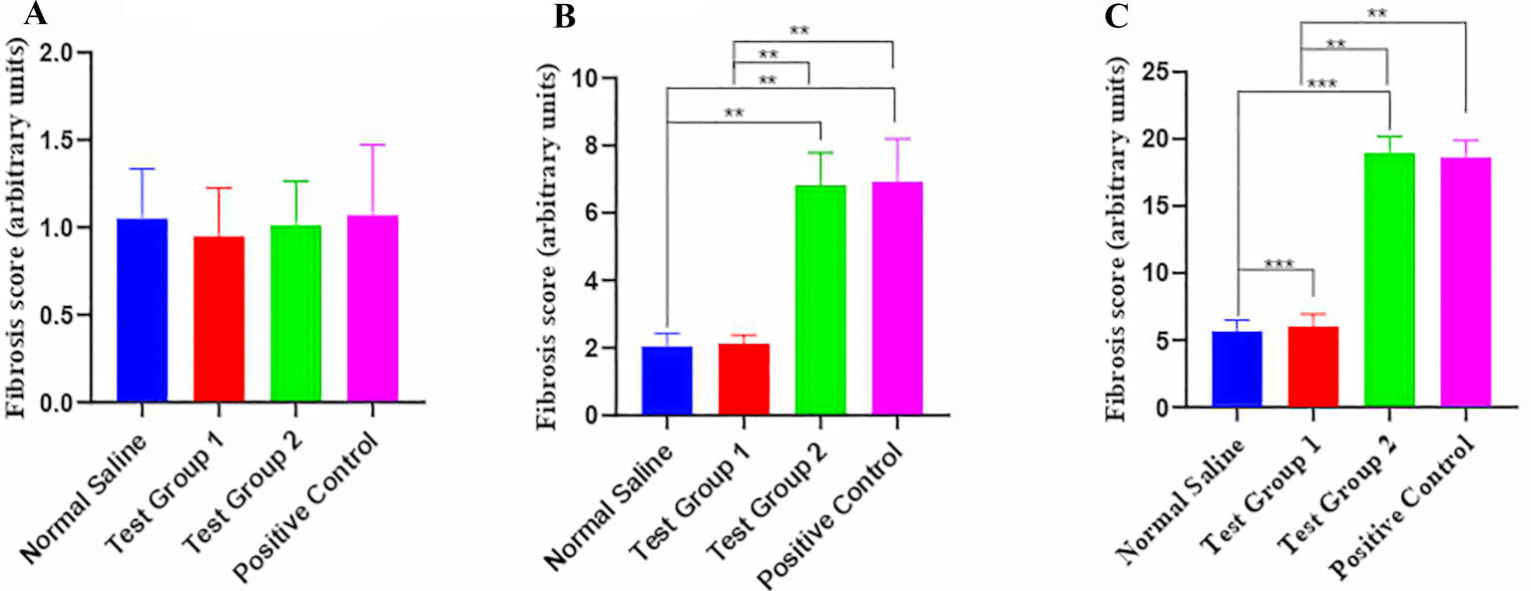
Quantification of fibrosis in the left ventricle of the three diet groups. **A**, Normal chow. **B**, Calorie-dense with normal protein diet. **C**, Calorie-dense with low protein diet. Data are reported as means±SE. **P<0.01, ***P<0.001; ANOVA. Standard group (SG): normal saline. Test group 1 (TG 1) - (DTG regimen + tesamorelin): Tenofovir disoproxil fumarate (TDF) + lamivudine (3TC) + dolutegravir (DTG) + tesamorelin. Test group 2 (TG 2) - (DTG regimen only): tenofovir disoproxil fumarate (TDF) + lamivudine (3TC) + dolutegravir (DTG). Positive control (PC) - (classical regimen): zidovudine (AZT) + lamivudine (3TC) + atazanavir/ritonavir (ATV/r).

**Figure 5:**
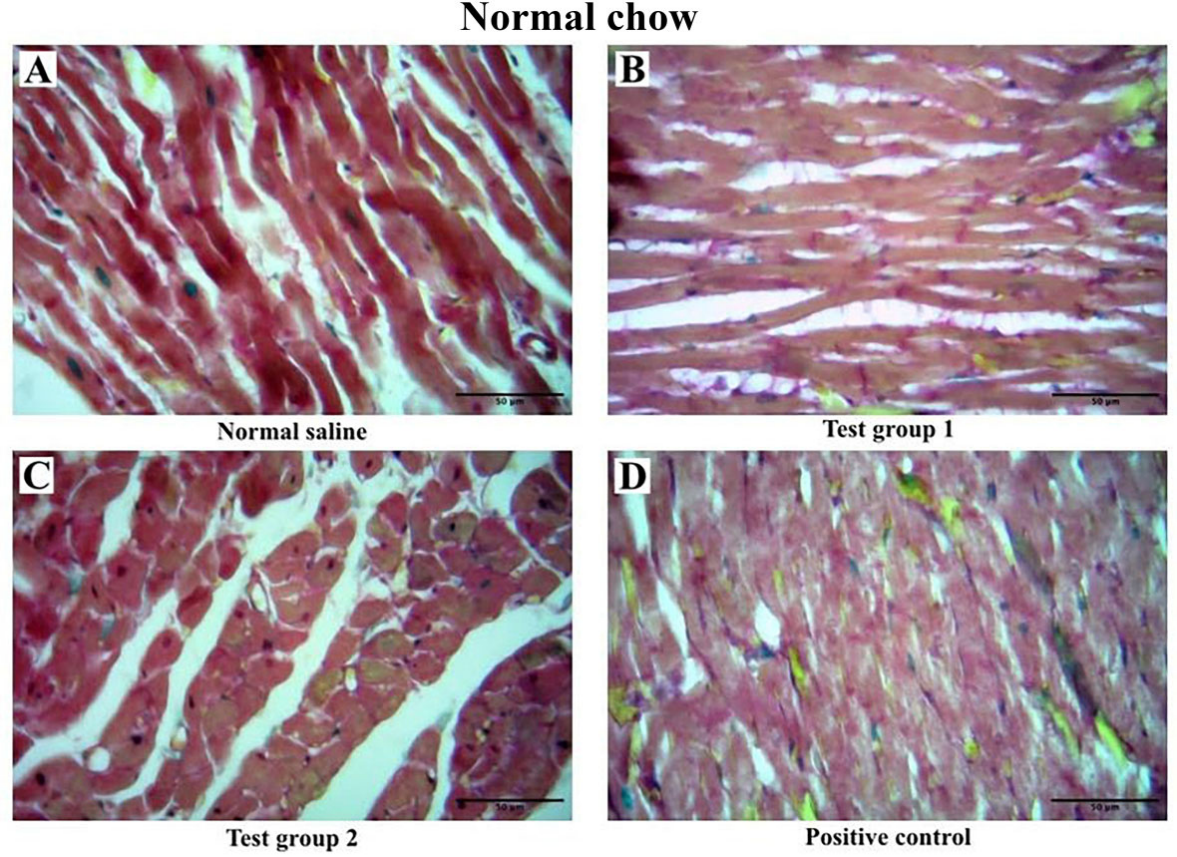
Representative histological cross-sections of the left ventricle from the normal chow diet group during the treatment phase. Sections were stained with Masson's trichrome, where collagen fibers appear green. Scale bar=50 μm. Images are representative of 10 rodents per group. Standard group (SG): normal saline. Test group 1 (TG 1) - (DTG regimen + tesamorelin): Tenofovir disoproxil fumarate (TDF) + lamivudine (3TC) + dolutegravir (DTG) + tesamorelin. Test group 2 (TG 2) - (DTG regimen only): tenofovir disoproxil fumarate (TDF) + lamivudine (3TC) + dolutegravir (DTG). Positive control (PC) - (classical regimen): zidovudine (AZT) + lamivudine (3TC) + atazanavir/ritonavir (ATV/r).

There was no variation in myocardial fibrosis among the four experimental groups. The graphical presentation of myocardial fibrosis and the histological sections of the left ventricle from the normal chow diet group at the end of the treatment phase are shown in Figures 4A and 5, respectively.

#### Calorie-dense with normal protein

**Figure 6:**
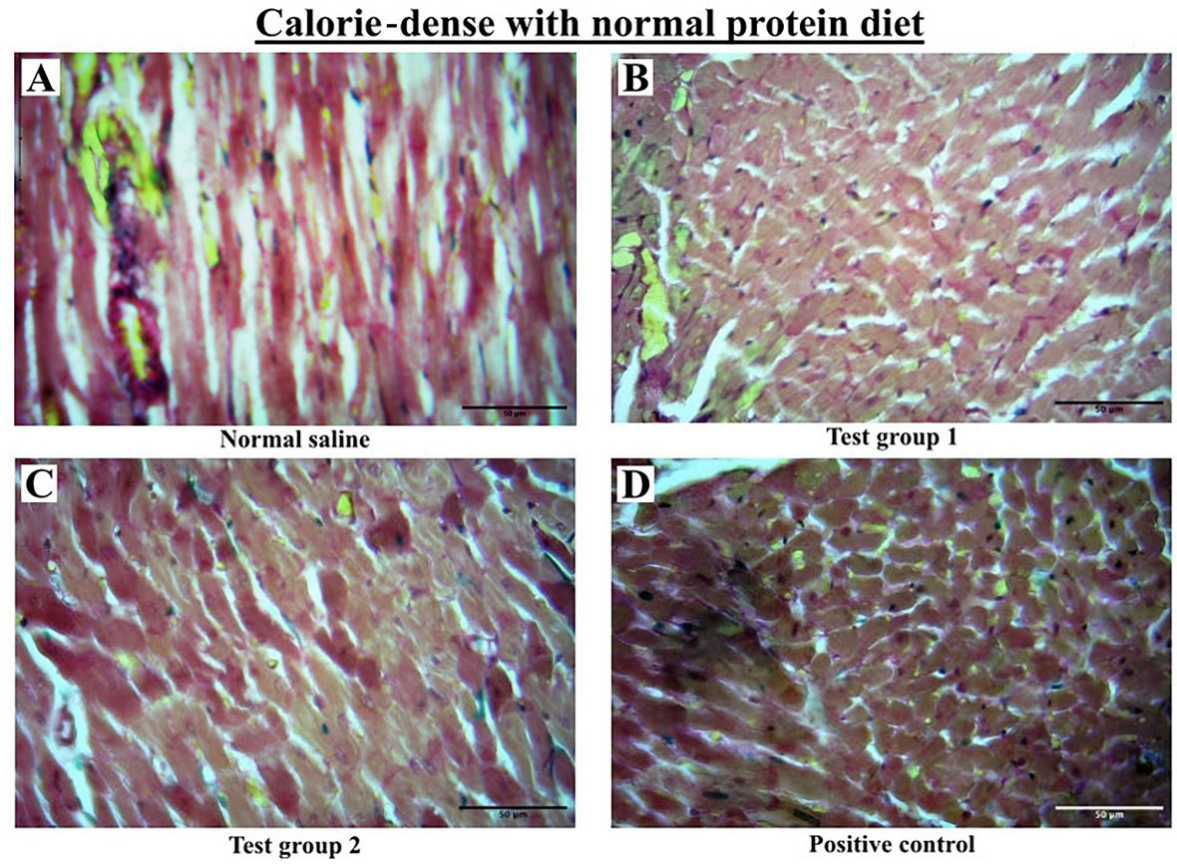
Representative histological cross-sections of the left ventricle from the calorie-dense with normal protein diet group during the treatment phase. Sections were stained with Masson's trichrome, where collagen fibers appear green. Scale bar=50 μm. Images are representative of 10 rodents per group. Standard group (SG): normal saline. Test group 1 (TG 1) - (DTG regimen + tesamorelin): Tenofovir disoproxil fumarate (TDF) + lamivudine (3TC) + dolutegravir (DTG) + tesamorelin. Test group 2 (TG 2) - (DTG regimen only): tenofovir disoproxil fumarate (TDF) + lamivudine (3TC) + dolutegravir (DTG). Positive control (PC) - (classical regimen): zidovudine (AZT) + lamivudine (3TC) + atazanavir/ritonavir (ATV/r).

There were significant variations in fibrosis among the four experimental groups. *Post hoc* statistical analysis using Dunn's *post hoc* comparisons test revealed significant differences between SG and TG 2 (P=0.0042), SG and PC (P=0.0034), TG 1 and TG 2 (P=0.0089), and TG 1 and PC (P=0.0073). The graphical presentation of myocardial fibrosis and the histological section of the left ventricle from the CDNP diet group at the end of the treatment phase are shown in Figures 4B and 6, respectively.

#### Calorie-dense with low protein

There was significant variation in fibrosis between the four experimental groups. *Post hoc* statistical analysis using Dunn's *post hoc* comparisons test revealed significant differences between SG and TG 2, SG and PC, TG 1 and TG 2, and TG 1 and PC (all P<0.001).

**Figure 7:**
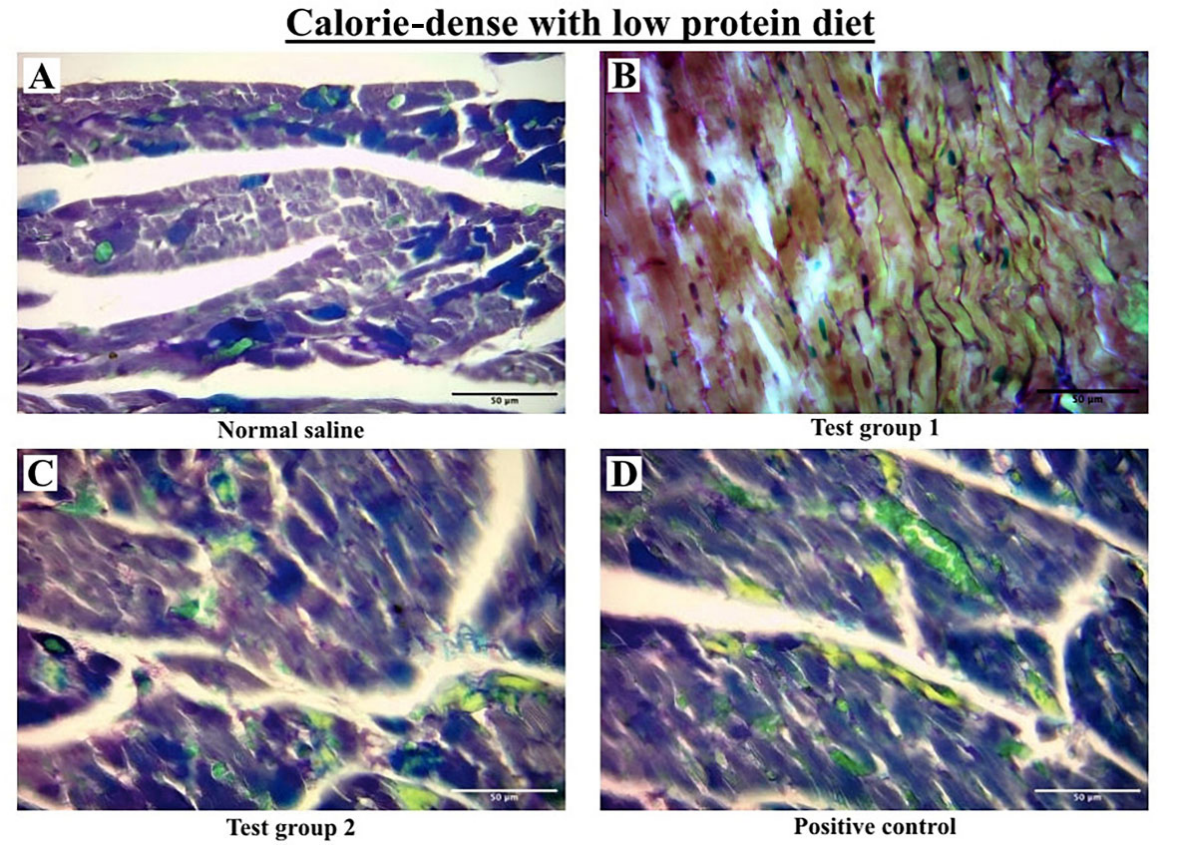
Representative histological cross-sections of the left ventricle from the calorie-dense with low protein diet group during the treatment phase. Sections were stained with Masson's trichrome, where collagen fibers appear green. Scale bar=50 μm. Images are representative of 10 rodents per group. Standard group (SG): normal saline; Test group 1 (TG 1) - (DTG regimen + tesamorelin): Tenofovir disoproxil fumarate (TDF) + lamivudine (3TC) + dolutegravir (DTG) + tesamorelin; Test group 2 (TG 2) - (DTG regimen only): tenofovir disoproxil fumarate (TDF) + lamivudine (3TC) + dolutegravir (DTG); Positive control (PC) - (classical regimen): zidovudine (AZT) + lamivudine (3TC) + atazanavir/ritonavir (ATV/r).

The graphical presentation of myocardial fibrosis and the histological section of the left ventricle from the CDLP diet group at the end of the treatment phase are shown in Figures 4C and 7, respectively.

## Discussion

The introduction of cART has led to decreased AIDS-related illness and death among individuals with HIV (11). However, the prevalence of age-related conditions like CVDs is increasing, emerging as the primary cause of mortality in this population (6). Recent studies have also indicated that HIV-positive individuals on cART have higher rates of CVDs such as myocardial infarction, stroke, sudden cardiac death, and heart failure, compared to those who are HIV-negative (12).

Low protein diets have been correlated with heart failure severity (13). A maternal diet low in protein results in permanent alterations in the structure and function (cardiac remodeling) of the developing heart of the fetus in humans, potentially increasing the likelihood of later onset cardiac abnormalities in the offspring (14).

We have previously shown that consumption of a calorie-dense diet with either normal or low protein content and intake of integrase-containing and classical cART drug regimens results in metabolic dysregulation in a rat model (15). The current study is the first to report that a DTG-containing and classical cART administered with a calorie-dense diet with either normal or low protein content results in ECG and cardiac histology changes in an animal model.

Metabolic syndrome (MetS) correlates with ECG abnormalities, possibly stemming from increased sympathetic system activity, elevation of the diaphragm, and increased cardiac output, resulting in left ventricular hypertrophy (16). Obesity may result in increased cardiac loading and remodeling of heart muscle, potentially causing ECG abnormalities (17). Also, endovascular impacts of obesity manifest through paracrine hormone expression of adipose tissue, potentially leading to alterations in atrial function (18).

Animals receiving both DTG-based and older cART regimens in the CDNP and the CDLP dietary groups had increased HR, which did not occur in the normal chow groups or the DTG plus tesamorelin group. The increase in HR and decrease in R-R interval may be attributed to the potentiation of the adrenergic overdrive in dysmetabolic states (metabolic syndrome) (19). Hyperinsulinemia due to dysmetabolic state is closely linked to increased sympathetic outflow and cardiac vagal withdrawal (20).

Both DTG-based and classical cART regimens led to a decrease in Q-wave amplitude. No decrease was observed in either the normal diet cohorts or the tesamorelin-treated group. The Q-wave amplitude represents the propagation of the impulse across the septum, and its reduction indicates a shift in the orientation of the septum leftwards. This alteration in septal positioning is observed in obese rats, where the heart tends to lie more horizontally within the chest cavity due to increased abdominal adiposity (21). This is a potential explanation for the observed experimental findings.

There was a significant increase in R-wave amplitude in animals receiving both DTG-only and older cART regimens, but no significant increase was found in either the normal chow group or the tesamorelin group. The R-wave, which is the positive deflection of the QRS complex, represents the depolarization of the anteroseptal region and the major part of the left ventricle (22). Higher values are suggestive of ventricular hypertrophy and significant coronary constriction (23). The increase in R-wave amplitude in cART-associated metabolic syndrome has not been previously documented in literature to the best of our knowledge.

There was prolongation of QTc interval in animals receiving DTG-based and classical cART regimens, but no significant prolongation was found in either the standard chow groups or the DTG plus tesamorelin group.

A prolonged corrected QT (QTc) interval serves as a predictor for mortality related to cardiovascular disease in both the overall population and those affected by HIV (24). Hence, the evaluation of the QTc interval has been suggested as a straightforward and noninvasive approach to assess cardiovascular risk in a clinical context (25). There are no previous reports, to the best of our knowledge, of QTc interval prolongation associated with DTG-based and classical cART regimens. Therefore, our study provides new evidence that DTG-containing therapy and classical cART administered with CDLP diet results in QTc interval prolongation in Sprague Dawley rats.

A prolonged QTc interval has been linked to myocardial electrical instability, contributing to adverse cardiovascular events such as ventricular fibrillation and sudden cardiac death (26).

QTc interval prolongation is attributed to insulin resistance, which is widely acknowledged to play a central role in the pathophysiology of MetS development (26). Insulin induces hyperpolarization in plasma membranes of both excitable and nonexcitable tissues, resulting in the prolongation of the QT interval (27). Hyperinsulinemia can also lead to hypokalemia, which in turn can cause QTc prolongation on an ECG (28).

Growth-hormone-releasing hormone (GHRH) has been demonstrated to enhance current flow through voltage-gated Ca^2+^ channels via the cAMP/PKA system (29). Additionally, GHRH reduces the current of voltage-gated K^+^ channels, engaging the protein kinase C (PKC) system in primary cultured ovine and human somatotropes (30). It has been proposed that GHRH enhances Na^+^ permeability through cAMP activation, resulting in cellular depolarization (31), and the subsequent opening of voltage-gated Ca^2+^ channels promotes the influx of Ca^2+^, leading to an elevation in intracellular [Ca^2+^] (30).

There were significant differences in the degree of left ventricular muscle fibrosis in CDLP and CDNP animals receiving both DTG-based and older cART regimens. It is notable that the effects of DTG on ventricular muscle tissue fibrosis were mitigated by co-administration of tesamorelin. Myocardial fibrosis resulting from excessive collagen deposition is the major contributor to left ventricular remodeling and heart failure in arrhythmogenic cardiomyopathy (ACM) (32). Myocardial fibrosis in ACM is characterized by both interstitial and perivascular fibrosis (33). cART has a cytotoxic effect on cardiac cells through inhibition of cardio-myocyte mtDNA leading to cellular injury and apoptosis. Prior data on adverse events of anti-HIV drugs suggested that the occurrence of myocardial infarction increased with extended exposure to cART (34).

Previously published studies have shown that GHRH analogues, i.e. MR-356, significantly reduce the infarct size and prevent ventricular remodeling (35), possibly by activating reparative pathways, such as AC/PKA and GC/PKG signaling, as seen with GHRH (36). An alternative theory is that chronic administration of growth hormone releasing hormone agonist (GHRH-A) during myocardial injury reverses ventricular remodeling and enhances cardiac performance, while also decreasing infarct size (37). GHRH-A increases expression of the transcription factor GATA-4 (38). It has been proposed that GATA-4 functions as an antiapoptotic factor necessary for adaptive responses, serving as a pivotal regulator of hypertrophy and hypertrophy-associated genes in the heart (39). Restoring diminished GATA-4 function reverses detrimental post-infarction remodeling by promoting myocardial angiogenesis, inhibiting apoptosis, and facilitating stem cell recruitment (40).

A key limitation of this study was that rodents were not HIV-positive, which limits the ability to fully replicate the cART-associated cardiac dysregulation observed in HIV-positive human subjects. Additionally, the study did not investigate the molecular mechanisms underlying the observed ECG changes and their recovery following tesamorelin treatment. Further research is needed to elucidate these pathways.

## Conclusions

This study demonstrated that both the CDNP diet (Western diet) and the CDLP diet, when combined with DTG-based or classical cART regimens, exert deleterious effects on cardiac effects, promoting myocardial fibrosis that disrupts normal electrical conduction and may predispose to arrhythmogenesis. Co-administration of tesamorelin successfully prevented ECG changes and fibrosis, indicating that growth hormone dysfunction may be involved in the underlying pathology.

## Data Availability

All data generated or analyzed during this study are included in this published article.
